# Proteomic Studies of Pathogenic Fungi and Hosts: A Multidisciplinary Approach to Therapeutics, Diagnostics, and Host Interactions

**DOI:** 10.3390/jof12090647

**Published:** 2026-09-01

**Authors:** Lucélia Santi, Walter Orlando Beys-da-Silva

**Affiliations:** Faculty of Pharmacy & Biotechnology Center, Federal University of Rio Grande do Sul, Porto Alegre 90610-000, RS, Brazil; walter.beys@ufrgs.br

Fungi constitute a highly diverse kingdom of organisms that are essential to ecosystems, agriculture, bioconversion, nutrition, biotechnology, and both human and animal health. However, fungal pathogens remain significant challenges and are a global public health concern, as demonstrated by the recent release of the World Health Organization’s Fungal Priority Pathogens List [1]. This list emphasizes species such as *Cryptococcus*, *Candida*, and *Aspergillus* due to their clinical relevance and increasing resistance to current therapies.

The Special Issue “Proteomic Studies of Pathogenic Fungi and Hosts” of the Journal of Fungi presents innovative research using proteomic technologies to deepen our understanding of fungal virulence, molecular interactions, and the host immune responses that shape disease outcomes. The contributions compiled here reflect the range and sophistication of contemporary proteomics in mycological research. These studies are applied not only to elucidate the complex molecular mechanisms by which fungi invade, persist, and evade host defenses but also to identify novel biomarkers and therapeutic targets essential for improving diagnostics and antifungal strategies. Key areas of focus include the characterization of extracellular vesicles, virulence-associated proteins, and host signaling pathways triggered during infection. By examining host-pathogen interactions at the proteome level, studies in this issue exemplify how molecular characterization can bridge fundamental biology and translational outcomes, particularly for *Cryptococcus* sp., *Paracoccidioides brasiliensis*, *Pneumocystis jirovecii*, *Coccidioides* sp., and phytopathogenic fungi such as *Aspergillus flavus* and *Alternaria alternata*. The development of precision diagnostics and next-generation antifungal treatments that can better treat invasive mycoses depends on these molecular insights.

Investigating extracellular vesicles (EVs) from virulent *P. brasiliensis*, Octaviano-Azevedo et al. [2] demonstrate that these structures selectively concentrate stress-response and virulence-associated proteins. Their findings distinguish the cargo of virulent EVs from those of attenuated variants and show how EV content is dynamically modulated by oxidative and nitrosative stress, providing new mechanistic insights into how fungal EVs facilitate pathogenesis. To address the challenge of data fragmentation, Calegari-Alves et al. [3] developed CRYPTOMICSDB, a specialized database that aggregates published molecular data on genes and proteins differentially expressed in both in vitro and in vivo infection models of *Cryptococcus*. Complementing this, the review by Betancourt and Nielsen [4] maps host-*Cryptococcus* protein networks and virulence mechanisms, offering a foundation for next-generation diagnostics and targeted therapies. Furthermore, Daniel et al. [5] provide an integrated computational-experimental framework that accelerates the discovery of virulence factors and prioritizes therapeutic targets in *Coccidioides*. The clinical implications of fungal colonization are also explored by Carmona-Pirez et al. [6], who present the first proteomic evidence of how *P. jirovecii* modulates molecular pathways in idiopathic pulmonary fibrosis (IPF), revealing metabolic and signaling signatures that advance our understanding of IPF pathogenesis.

Beyond human health, this Special Issue addresses the critical field of plant pathology. A review by Corrêa et al. [7] summarizes how mass spectrometry-based proteomics has revolutionized the study of phytopathogenic fungi (including *A. flavus*, *A. alternata*, *Fusarium* spp., and *Botrytis cinerea*). This work reveals molecular adaptations to stress and mechanisms of antifungal resistance, guiding the development of sustainable strategies for plant disease control.

Collectively, this Special Issue highlights how the integration of advanced proteomics, computational modeling, and curated molecular resources can overcome traditional bottlenecks in the study of high-priority and emerging fungal pathogens. By combining predictive in silico frameworks with accessible databases, these works provide a strategic roadmap for translating complex molecular knowledge into improved diagnostics, novel drug targets, and effective vaccines for life-threatening diseases such as coccidioidomycosis, pneumocystosis, and cryptococcosis.

We thank all authors and reviewers who contributed their expertise to this issue. Their pioneering research reinforces the value of proteomics as a cornerstone of modern mycology and highlights the ongoing need for integrative, multi-omics strategies to combat the global burden of fungal disease. We hope this collection inspires further investigations that unravel the proteomic complexity of host-fungal interactions and accelerate the translation of fundamental discoveries into clinical and agricultural innovations.

## Data Availability

No new data were created or analyzed in this study. Data sharing is not applicable to this article.
